# Facial Nerve Palsy in a Five-Month-Old Infant

**DOI:** 10.7759/cureus.39799

**Published:** 2023-05-31

**Authors:** Amber Vozar, John Dugas, Seth J Deskins, Sharda Udassi

**Affiliations:** 1 Pediatrics, West Virginia University, Morgantown, USA; 2 Internal Medicine and Pediatrics, West Virginia University Medicine, Morgantown, USA

**Keywords:** bell's palsy diagnosis, neurology, pediatrics, cranial nerve 7, cranial nerve palsy (cnp), facial nerve

## Abstract

Facial nerve palsy is a common neurological disorder, and the etiology is categorized as either congenital or acquired. Even after extensive workup, a vast majority of cases are deemed idiopathic. Treatment of acquired facial nerve palsy in pediatrics is essential to prevent long-term aesthetic and functional complications. The prognosis is favorable in pediatric patients and those treated with corticosteroids.

## Introduction

Cranial nerve seven (facial nerve) palsy is one of the most common neurological disorders in children and is commonly referred to as Bell’s palsy when the etiology is unknown [1]. It is estimated that the yearly incidence of Bell’s palsy is 6.1 per 100,000 cases in patients aged between one and 15 years, and even less frequent in infancy [2]. Approximately half the cases of facial palsy are idiopathic [3]. Prognosis varies, but a large proportion of patients experience spontaneous resolution. Pediatric patients typically have more favorable outcomes compared to adult patients [3,4]. Treatment focuses on both treating the underlying etiology and minimizing long-term sequelae. Most patients are treated with corticosteroids of varied doses and duration. Here, we present a case of facial nerve palsy in a five-month-old infant.

## Case presentation

**Figure 1:**
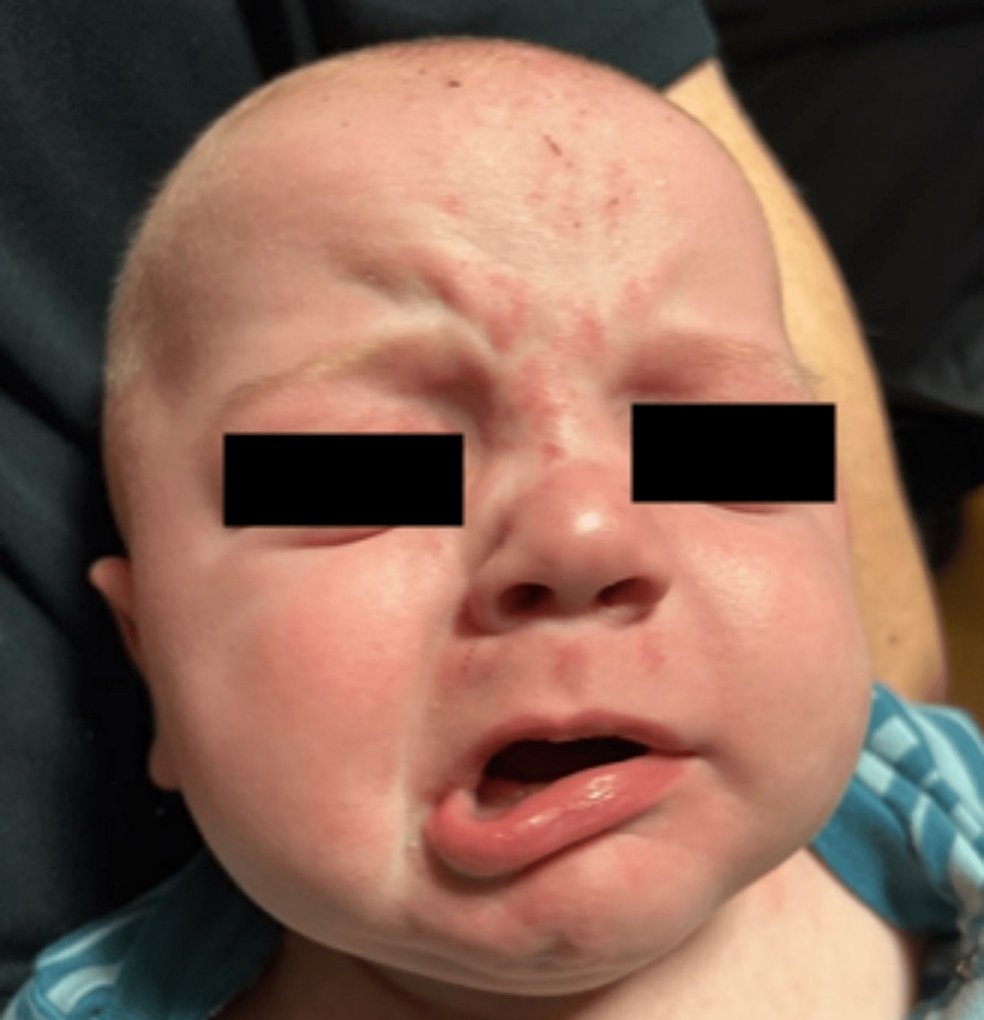
Left facial paresis. The infant shows incomplete closure of the left eye and buccal border deviation to the right (paretic left labial angle).

A five-month-old male with no past medical history was admitted for left-sided facial droop. The patient was seen one day prior to admission at his primary care provider’s office for cough, nasal congestion, rhinorrhea, and low-grade fever. Workup was remarkable for a respiratory syncytial virus (RSV) on a respiratory viral panel. The following day, he developed a left-sided facial droop with decreased wrinkles on the left side of his forehead (Figure 1). 

The development of left-sided facial droop prompted admission for further workup. Further history was obtained upon admission and revealed that the patient was born at 38 weeks via low transverse caesarian section due to failure to progress with one-minute and five-minute Apgar scores of 8 and 9, respectively. The maternal screening was unremarkable and included negative testing for group B streptococcus, human immunodeficiency virus, syphilis, hepatitis B and C, gonorrhea/chlamydia, and the mother’s rubella status was immune. Her pregnancy was only complicated by well-controlled anxiety/depression and a history of herpes simplex virus (HSV), for which she was compliant with Valtrex therapy. Of note, there were no active documented herpetic lesions during pregnancy. As noted, on arrival at our facility, the patient had a left-sided facial nerve palsy with incomplete closure of the left eye, and his buccal rim deviated to the right (left labial angle paretic) (Figure 1). Pupils were equal, round, and reactive to light, without lesions on or surrounding the eye. The remaining cranial nerves were intact, and his physical exam was otherwise unremarkable including normal external ear and tympanic membrane. 

Upon admission, laboratory workup was significant for an increased white blood cell count, platelets, C-reactive protein, and erythrocyte sedimentation rate (Table 1). Repeat values were not obtained.

**Table 1:** Elevated laboratory values upon admission with reference ranges

Laboratory data	Values at admission	Reference ranges
White blood cell count	15.5	6.5-13.3 x 10^3/uL
Platelets	595	244-529 x10^3/uL
C-reactive protein	86.9	<8 mg/L
Erythrocyte sedimentation rate	34	0-15 mm/hr

A lumbar puncture was performed, and cerebrospinal fluid (CSF) studies were all normal, having two nucleated cells, protein of 21, and a glucose of 58. Other negative CSF tests included enterovirus, arbovirus, and HSV polymerase chain reaction. The basic metabolic panel, hepatic function panel, and procalcitonin were all within normal limits. Blood, urine, and CSF cultures all showed no growth. Magnetic resonance imaging (MRI) with contrast of the brain was performed and revealed no cerebral lesions or evidence of ischemic events. The patient’s symptoms resolved over a 48-hour period without intervention. He was discharged home with outpatient neurology follow-up. At the one-month follow-up, the patient was healthy without any neurological deficits or signs of recurrent facial nerve palsy. The patient's audiology testing, performed as an outpatient, was normal.

## Discussion

Cranial nerve seven palsy is one of the most common neurological disorders seen in children, but it is less common in infants. Known etiologies for infantile facial nerve palsy are divided into congenital (delivery traumas and genetic abnormalities) and acquired (infectious, inflammatory, neoplastic, and traumatic) categories [3,4].

The prognosis can be difficult to assess for facial paralysis in children; however, in approximately 70% of cases, there is spontaneous resolution. Complete functional recovery is also more likely in pediatric patients compared to adults [4]. The House-Brackmann scale is a tool used to rate the severity of paralysis. Degree II (mild dysfunction) generally has good outcomes, while degrees III and IV (moderate and severe dysfunction, respectively) and V and VI (complete paralysis) are associated with poorer prognoses [5,6]. A favorable prognostic indicator for resolution is clinical improvement within three weeks of onset [6]. Ramsay Hunt syndrome has one of the worst prognoses for facial nerve palsy, with only 10% of severe paralysis from herpes varicella-zoster having complete resolution [7].

Treatment of facial nerve palsy is dependent on the etiology and severity. However, due to the rarity of idiopathic facial palsy in infants, some documented cases were preceded by a symptomatic upper respiratory infection (URI) prior to or during the onset of facial paralysis. As no specific pathogen was identified for the URI, these cases were deemed to be idiopathic palsies despite the prodromal or concurrent illnesses [1], as in our case, where no standardized treatment regimen has been established for this age group. In a recent case report of grade IV Bell’s palsy in a neonate, six weeks of steroids were used [5], but even short-term steroid use in the neonatal period is associated with significant potential for harm, with adverse effects ranging from hypertension, hyperglycemia, hypertrophic cardiomyopathy, and growth failure. There are limited clinical trials for the treatment of Bell’s palsy in children, and given its favorable prognosis in this patient population, the goal of therapy is to minimize incomplete resolution and the risk of sequelae, such as autonomic dysfunction, facial spasms, and synkinesis [8]. 

For pediatric patients, it is recommended to begin oral corticosteroids within three days of onset at 1 ml/kg to 2 ml/kg per day with a prolonged taper [6]. The majority of patients typically respond within three weeks; however, several studies did not find significant differences in outcomes for children who were not treated with steroids [9]. In contrast, patients with Ramsay Hunt syndrome are required to be treated with intravenous steroids and antivirals as soon as possible to garner the best prognosis. There have also been advancements in surgical, rehabilitation, and even regenerative therapies for cases refractory to glucocorticoids and anti-viral therapies [10].

## Conclusions

Here, we document a case of idiopathic cranial nerve seven palsy in infancy. It is very rare for idiopathic facial palsy to occur in infants; thus, a thorough workup is required. Workup should include a thorough birth and infectious disease history, including screening for Ramsay Hunt syndrome to guide treatment. Given the potential hindrance that severe facial palsy poses for breastfeeding, we suggest consideration of steroid therapy in patients with a shorter total duration after the risk/benefit discussion. In this case, the facial nerve resolved without intervention, and thus steroids were not initiated. 
